# Metabolic/hypoxial axis predicts tamoxifen resistance in breast cancer

**DOI:** 10.1038/s41598-022-19977-w

**Published:** 2022-09-27

**Authors:** Hany N. Azzam, Marwa O. El-Derany, Sara A. Wahdan, Reham M. Faheim, Gouda K. Helal, Ebtehal El-Demerdash

**Affiliations:** 1 Department of Pharmacology and Toxicology, Faculty of Pharmacy, Heliopolis University, Cairo, Egypt; 2 Department of Biochemistry, Faculty of Pharmacy, Ain Shams University, Cairo, Egypt; 3 Department of Pharmacology and Toxicology, Faculty of Pharmacy, Ain Shams University, Cairo, Egypt; 4 Department of Clinical Oncology and Nuclear Medicine, Faculty of Medicine, Ain Shams University, Cairo, Egypt

**Keywords:** Molecular biology, Oncology

## Abstract

We sought in our cross-sectional study to investigate the role of metabolic/hypoxial axis in the development of tamoxifen (TMX) resistance in BC patients. Quantification of plasma LncRNA Taurine upregulated-1 (TUG-1), miRNA 186-5p (miR-186), serum Sirtuin-3 (SIRT3), Peroxisome Proliferator Activator Receptor alpha (PPAR-1 α) and Hypoxia Inducible Factor-1 (HIF-1α) was done in a cohort of patients divided into TMX-sensitive and TMX-resistant candidates. Multiple logistic regression and Receiver Operating Characteristic curve were developed for significant predictors. Plasma TUG-1 and miR-186 were significantly elevated in TMX resistant patients. Serum proteins SIRT3, PPAR-1 α and HIF-1α were deficient in TMX resistant patients compared to TMX sensitive patients, respectively. miR-186 was associated with respiratory symptoms, while, HIF-1α was associated with metastases in TMX resistant patients. Strong correlations were found between all parameters. A predictive model was constructed with TUG-1 and HIF-1α to estimate TMX resistance in BC patients with 88.3% sensitivity and 91.6% specificity. Hypoxia and metabolic dysregulations play important role in the development of TMX resistance in BC patients. Correlation between hypoxia, carcinogenesis and patient’s mortality have led to more aggressive phenotypes, increased risk of metastasis and resistance to TMX.

## Introduction

Tamoxifen (TMX) treatment of estrogen (ER) positive breast cancer (BC) reduces mortality by 31%. However, over half of advanced ER-positive breast cancers are intrinsically resistant to TMX and about 40% will acquire the resistance during the treatment^1^.

Long-noncoding RNAs (LncRNA) are a specific type of ncRNA, which play a role in apoptosis^2^, differentiation^3^, protein degradation and cell proliferation^4^. Taurine upregulated 1 (TUG-1) is a novel lncRNA that has been engaged with human cancer^5^. In particular, TUG-1 shows high potential to exacerbate toxic side effects of classic chemotherapeutic drugs such as TMX, thereby upgrading their therapeutic efficacy^6^. Dysregulation of TUG-1 has been expressed with proliferation, migration, cell cycle changes, inhibited apoptosis, and drug resistance of cancer cells, which manifested an oncogenic role for this lncRNA^7^.

Recent studies showed TUG-1 to function as a sponge for miRNA 186-5p (miR-186). In fact, decreased expression of miR-186 was also reported in BC^8,9^. Dysregulation of micro RNAs (miRNAs) not only affect cellular processes involved in carcinogenesis but can reform therapeutic interventions, as recent studies have highlighted. In BC, well-studied oncogenic miRNAs have been shown to revitalize chemoresistance in vitro through their synchronization of key resistance-associated proteins^10^. Interestingly, Sirtuin3 (SIRT3), NAD (+)-dependent deacetylases, was introduced as a new presenter of TMX resistance in BC cells^11^. Whereas, SIRT3 has been described as a tumor suppressor in BC^12^.

Cancer cells have evolved the adeptness to reprogram their cellular metabolism to encourage growth and tumor progression by regulation of the Warburg effect and tumorigenesis^13^. Metabolically, SIRT3 was proved to be a new target of peroxisome proliferator activator receptor alpha (PPAR-1α)^14^. In the same line, interesting association was established between SIRT3 and hypoxia through hypoxia inducible factor 1α (HIF-1α), transcription factor, in cancer^15^. Actually, HIF-1α directs multiple functions in cells such as metabolism, survival, proliferation, apoptosis, migration, energetic balance, and pH^16^.

Seeking an early diagnostic marker is ongoing, where non-invasive strategies for predicting TMX resistance are of crucial effect, in order to diminish mortality and enhance quality of life. Accordingly, we aimed to investigate and explore the possible role of TUG-1/miR-186/SIRT3, PPAR-1α and HIF-1α axis in TMX resistance and TMX sensitive BC patients and to correlate the aforementioned axis with tumor progression, different clinical presentations, anthropometric parameters and other metabolic biomarkers in BC. Consequently, of concern, this might provide an attractive strategy to find a diagnostic marker and to overcome TMX resistance for better BC treatment.

## Subjects and methods

### Subject recruitment and study design

This study was approved by the Ethical Committee of faculty of pharmacy and faculty of medicine, Ain Shams University, Cairo, Egypt, under Federal Wide Assurance approval (FWA). It was performed in adherence to the Declaration of Helsinki Guidelines. Written informed consent was signed by all study subjects after being informed about the investigations, study protocol, potential risks, purpose and benefits of the study. A complete medical history, physical examination and demographic information was obtained for all participants.

Those participants were histologically confirmed, diagnosed and pathologically staged BC patients at oncology outpatient clinics, Al Demerdash hospital, Ain Shams University, and were classified into the following two groups under the supervision of a professional oncologist:

1. **Group 1:** TMX-sensitive BC patients (n = 50).
2. **Group 2:** TMX-resistant BC patients (n = 50).

Exclusion criteria for BC patients included the following (a)—patients with other cancer types. (b)—Male gender, (c)—patients taking medications for any metabolic diseases (chronic renal disease, liver disease, hyper/hypothyroidism, diabetes Mellitus (DM), malabsorption syndrome etc.,), (d)—pregnant or breast-feeding women, (e)—patients taking anticonvulsants, glucocorticoids or immunosuppressant drugs, (f)—any other medical or physical abnormality, or disorder that would prohibit the patient from completing study procedures in the judgment of the investigator.

Five milliliters (mL) of venous blood samples were collected from an antecubital vein in the sitting position after 10 min rest. Blood samples were divided into 2 vacutainers one plain vacutainer for serum preparation and one K2-EDTA vacutainer for plasma preparation. Plain vacutainers were allowed to clot for 30 min. Plain and K2-EDTA vacutainers were then centrifuged at 4000 round per minute (r.p.m) for 10 min. The obtained serum and plasma were aliquoted and kept at − 80 °C for subsequent use.

### Extraction and quantification of non-coding RNA using the mirVANA PARIS kit

The extraction of non-coding RNA was performed using mirVANA PARIS commercially available kit supplied from Applied Biosystems according to manufacturer’s instructions (*Part Number AM1556*)^17,18^. The concentration and purity of RNA solutions was determined by measuring their absorbance at 260 and 280 nm, using the NanoDrop 1000A Spectrophotometer for spectrophotometer readings. This was done by measuring 1.5 μL of each RNA sample directly. RNA readings were measured in ng/μL.

### Quantification of LncRNA (TUG-1) and miRNA (miR-186) by RT-PCR

Quantification of LncRNA (TUG-1) was performed using commercially available Taqman kit supplied from Applied Biosystems according to manufacturer’s instruction*s *(*Part Number 4375575*)^19–21^. The process of qt-PCR was started and results were displayed from the cDNA for 2 different gene targets; TUG-1 and its housekeeping control Glyceraldehyde-3-phosphate dehydrogenase (GAPDH). The threshold cycle^22^ values were calculated for both target genes samples (TUG-1 and GAPDH). Delta CT values, Delta (delta CT) values and induction percentages values were calculated.

Quantification of miR-186 was performed using commercially available Taqman kit supplied from Applied Biosystems according to manufacturer’s instructions (*Part Number 4364031*)^23–26^. The process of qt-PCR was started and results were displayed from the cDNA for 2 different gene targets; miR-186 and its housekeeping control U6. The CT values were calculated for both target genes samples (miR-186 and U6). Finally, Delta CT values, Delta (delta CT) values and induction percentages values were calculated.

### Assessment of human serum levels of SIRT3

The concentration of human serum SIRT3 was determined by ELISA technique using commercially available kit supplied from Bioassay Technology Laboratory (China) according to manufacturer’s instructions (*Cat no. E2559Hu*)^27,28^.

### Assessment of human serum levels of PPAR-1 α

The concentration of human serum PPAR-1 α was determined by ELISA technique using commercially available kit supplied from Bioassay Technology Laboratory (China) according to manufacturer’s instructions (*Cat no. E1450Hu*)^29,30^.

### Assessment of human serum levels of HIF-1α

The concentration of human serum HIF-1α was determined by ELISA technique using commercially available kit supplied from Bioassay Technology Laboratory (China) according to manufacturer’s instructions (*Cat no. E0422Hu*)^31,32^. The results of serum **SIRT3, PPAR-1 α** and **HIF-1α** were calculated automatically using a linear regression equation of a standard curve, which was constructed by plotting absorbance value for each sample on the Y-axis versus its corresponding concentration on the X-axis, where **SIRT3, PPAR-1 α** and **HIF-1α** concentration was determined from such standard curves.

### Statistical analysis

Data were collected, revised, coded and entered to the Statistical Package for Social Science (IBM SPSS) version 23. The quantitative data was presented as mean, standard deviations and ranges when parametric and median with inter-quartile range (IQR) when non parametric. Also, qualitative variables were presented as numbers and percentages. The comparison between two independent groups with quantitative data and parametric distribution was done by using independent t-test while that with non-parametric distribution were done by using Mann–Whitney test. Spearman correlation coefficients were used to assess the correlation between two quantitative parameters in the same group. ROC curve was used to assess the best cut off point for predictors of mortality with its sensitivity, specificity, positive predictive value, negative predictive value (NPV) and area under curve (AUC). Logistic regression analysis (univariate and multivariate) was used to assess the association between symptoms and different parameters with its odds ratio and 95% CI. Kaplan–Meier survival analysis was used to assess the relation between different parameters and survival (months) and also to compare between resistant and sensitive groups using Log Rank test. The confidence interval (CI) was set to 95% and the margin of error accepted was set to 5%. The threshold for statistical significance was set at P < 0.05 for all statistical tests^33^.

## Results

### Clinical characteristics of TMX-sensitive and TMX-resistant patients

A total of hundred patients of the same race and ethnicity were enrolled in this study. Fifty Egyptian subjects were TMX-sensitive, while the other fifty were TMX-resistant. Our subjects were age and body mass index (BMI) matching. No significant difference was found between sensitive and resistant groups regarding age and BMI. For example, age mean ± standard deviation was 46.96 ± 6.51 in the TMX-sensitive group compared to 44.90 ± 8.44 in the TMX-resistant group. Furthermore, no significant difference was found between Group A and Group B patients with respect to the routine blood analysis, liver functions and serum creatinine (*P* value > 0.05). For instance, red blood cells (RBCs) mean ± standard deviation was 4.1 ± 0.3 in the TMX-sensitive group compared to 4.3 ± 0.4 in the TMX-resistant group.

### Association of TUG-1/miRNA 186-5/SIRT3, PPAR-1α and HIF-1α with TMX resistance

Plasma median levels of TUG-1 and miR-186 were significantly elevated in TMX resistant patients by 5.3-fold change and 2.2-fold change, respectively, in comparison to age-matched TMX sensitive patients. Whereas, serum median levels of SIRT 3, PPAR-1 α and HIF-1 α were significantly deficient in TMX resistant patients by 1.3-fold change, 1.5-fold change and 1.6-fold change, respectively, in comparison to age-matched TMX sensitive patients. This was shown clearly in Table 1.

**Table 1:** Comparison between TMX sensitive and TMX resistant groups regarding the different studied parameters.

	TMX-sensitive group	TMX-resistant group	Test value	P value
	N = 50	N = 50		
**TUG-1**				
Median (IQR)	39.75 (32.86–45.99)	210.62 (172.64–220.57)	7.127	< 0.001**
Range	23.03–136.35	36.60–448.22		
**miRNA 186-5p**				
Median (IQR)	168.78 (138.51–180.81)	376.08 (339.77–428.06)	5.933	< 0.001**
Range	111.73–568.78	111.07–620.37		
**SIRT3**				
Median (IQR)	3.57 (2.55–4.54)	2.85 (2.14–3.47)	− 2.642	0.008**
Range	0.46–9.78	0.38–5.69		
**PPAR alpha**				
Median (IQR)	1.21 (0.75–2.77)	0.83 (0.64–1.05)	− 2.902	0.004**
Range	0.29–4.56	0.35–2.92		
**HIF-1**				
Median (IQR)	1.99 (1.73–2.95)	1.23 (0.99–1.57)	− 5.108	0.001**
Range	0.45–5.72	0.53–3.97		

P value > 0.05: non significant; P value < 0.05: significant*; P value < 0.01: highly significant**.

^≠^Mann–Whitney test.

### Most common symptoms of TMX resistance among our studied cohort

**Figure 1:**
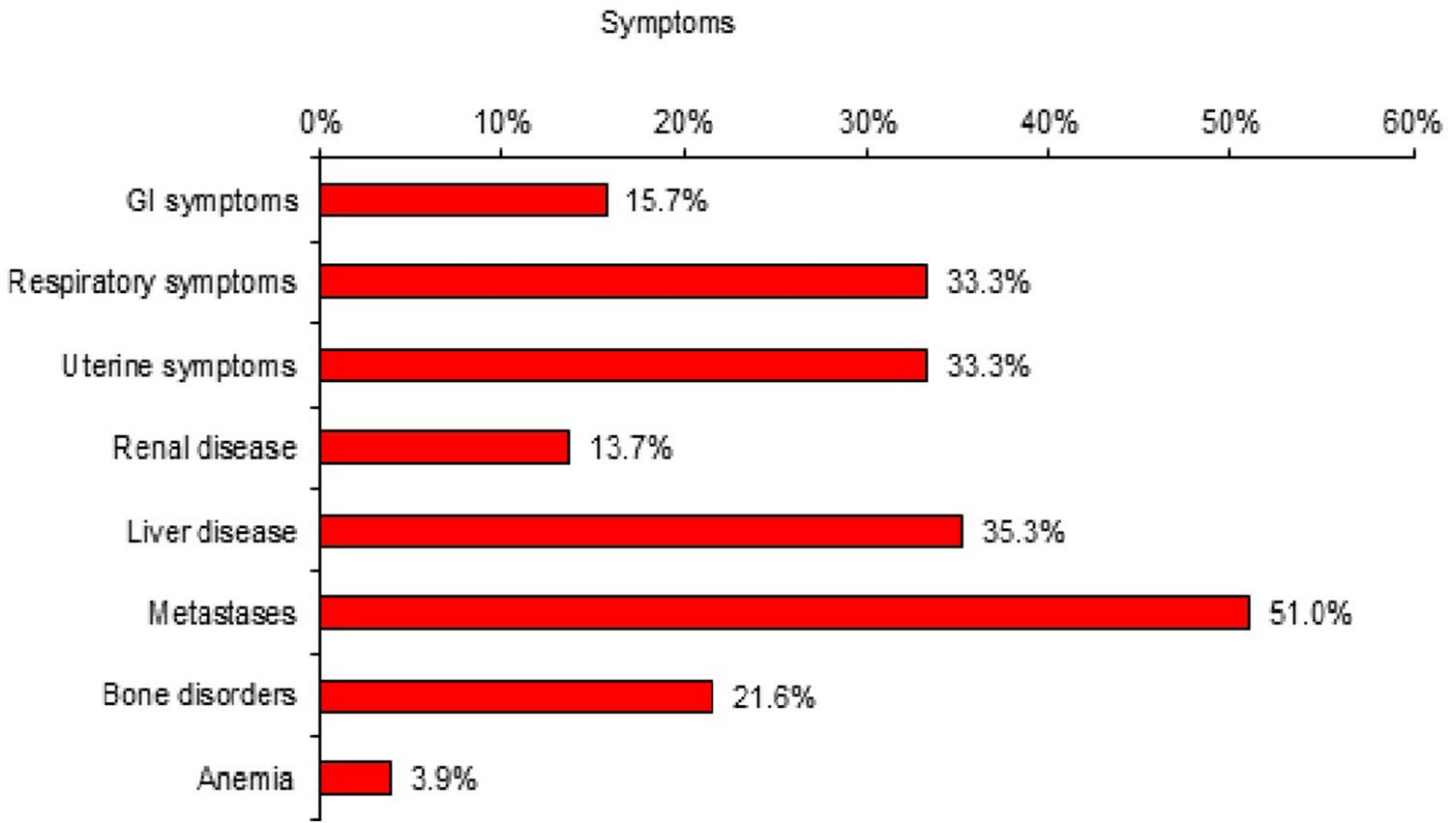
Symptoms for TMX resistance among the studied TMX-resistant group. Several symptoms were responsible for stopping TMX and/or shifting to another drug for the patients in the TMX resistant group. The red bars in this figure shows 8 of the most dominant symptoms. Metastases was the most predominant symptom (51%) followed by liver disease (35.3%) and respiratory symptoms (33.3%).

Several symptoms were responsible for stopping TMX and/or shifting to another drug for the patients in the TMX resistant group. Metastases was the most predominant symptom (51%). Moreover, respiratory symptoms compromised 33.3%. This is shown clearly in Fig. 1.

### Association between TUG-1/miRNA 186-5/SIRT3, PPAR-1α, HIF-1α and symptoms of TMX resistance

**Figure 2:**
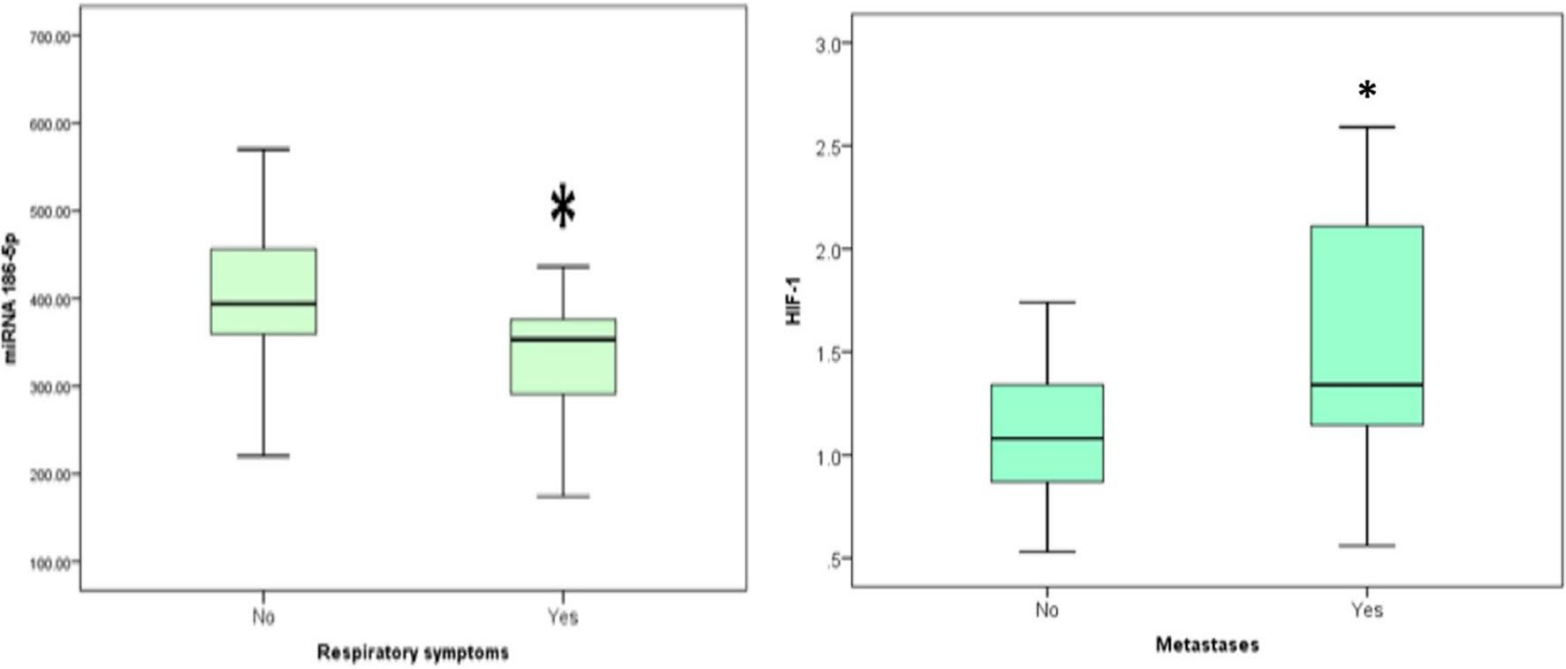
The boxplot (median and IQR) for the plasma miR-186 levels with TMX-resistant patients having respiratory symptoms and for the serum HIF-1α levels with TMX-resistant patients having metastases. In this figure, Mann–Whitney test was used to compare two independent groups with quantitative data and non-parametric distribution. This box plot figure shows a significant negative association between miR-186 and respiratory symptoms at test value of − 2.376 and P value of 0.018. It also shows a significant positive association between HIF-1 α and metastases at test value of − 2.224 and P value of 0.026. Logistic regression analysis showed that there was statistically significant association found between miR-186 ≤ 376.08 and respiratory symptoms with P value = 0.005 and OR (95% CI) of 8.667 (1.904–39.442), where respiratory symptoms rate increase as miR-186 decreases in TMX resistant patients. It also showed that there was statistically significant association found between HIF-1α > 1.12 and metastases with P value = 0.009 and OR (95% CI) of 6.171 (1.583–24.054), where metastases rate increase as HIF-1α increases in TMX resistant patients.

There was no statistical significance association found between all TMX-resistance symptoms and the different studied markers, except for miR-186, which showed negative association with respiratory symptoms at test value of − 2.376 and P value of 0.018 as shown in Table 2 and Fig. 2. Besides, HIF-1α showed positive association with metastases at test value of − 2.224 and P value of 0.026 as shown in Table 3 and Fig. 2. Logistic regression analysis showed that there was statistically significant association found between miR-186 ≤ 376.08 and respiratory symptoms with P value = 0.005 and OR (95% CI) of 8.667 (1.904–39.442), where respiratory symptoms rate increase as miR-186 decreases in TMX resistant patients. It also showed that there was statistically significant association found between HIF-1α > 1.12 and metastases with P value = 0.009 and OR (95% CI) of 6.171 (1.583–24.054), where metastases rate increase as HIF-1α increases in TMX resistant patients.

**Table 2:** Relationship between respiratory symptoms and the different studied parameters in TMX-resistant group.

	Respiratory symptoms		Test value	P value
	No	Yes		
	No. = 34	No. = 16		
**TUG-1**				
Median (IQR)	211.11 (173.58–219.81)	394.05 (359.39–456.47)	− 0.335^≠^	0.737
Range	36.60–267.36	220.38–570.02		
**miR-186**				
Median (IQR)	394.05 (359.39–456.47)	353.22 (290.82–376.08)	− 2.376^≠^	0.018*
Range	220.38–570.02	111.07–620.37		
**SIRT3**				
Median (IQR)	3.04 (2.14–3.72)	2.68 (2.11–3.47)	− 0.551^≠^	0.581
Range	0.38–4.92	1.33–5.69		
**PPAR alpha**				
Median (IQR)	0.83 (0.64–1.05)	0.95 (0.54–1.21)	− 0.538^≠^	0.590
Range	0.35–2.48	0.4–2.92		
**HIF-1**				
Median (IQR)	1.23 (0.95–1.57)	1.19 (0.99–1.74)	− 0.039^≠^	0.969
Range	0.53–2.3	0.54–3.97		

P value > 0.05: non significant; P value < 0.05: significant*; P value < 0.01: highly significant**.

Significant values are in [bold].

^≠^Mann–Whitney test.

**Table 3:** Relationship between metastases and the different studied parameters in TMX-resistant group.

	Metastases		Test value	P value
	No	Yes		
	No. = 24	No. = 26		
**TUG-1**				
Median (IQR)	210.12 (173.58–218.17)	212.61 (171.69–228.52)	− 0.835^≠^	0.404
Range	36.6–243.83	44.14–448.22		
**miR-186**				
Median (IQR)	376.08 (339.77–419.89)	376.08 (311.07–456.08)	− 0.041^≠^	0.967
Range	256.85–466.08	111.07–620.37		
**SIRT3**				
Median (IQR)	2.79 (1.92–3.47)	3.04 (2.39–3.72)	− 0.784^≠^	0.433
Range	0.5–4.92	0.38–5.69		
**PPAR alpha**				
Median (IQR)	0.81 (0.54–0.95)	0.85 (0.67–1.21)	− 1.289^≠^	0.197
Range	0.4–1.82	0.35–2.92		
**HIF-1**				
Median (IQR)	1.08 (0.79–1.37)	1.34 (1.13–2.13)	− 2.224^≠^	0.026*
Range	0.53–1.74	0.56–3.97		

P value > 0.05: non significant; P value < 0.05: significant*; P value < 0.01: highly significant**.

Significant values are in [bold].

^≠^Mann–Whitney test.

### Kaplan–Meier analysis for comparison between TMX-sensitive and TMX-resistant groups regarding overall survival (months) and for the relationship between TUG-1 and miR-186 with survival (months)

**Figure 3:**
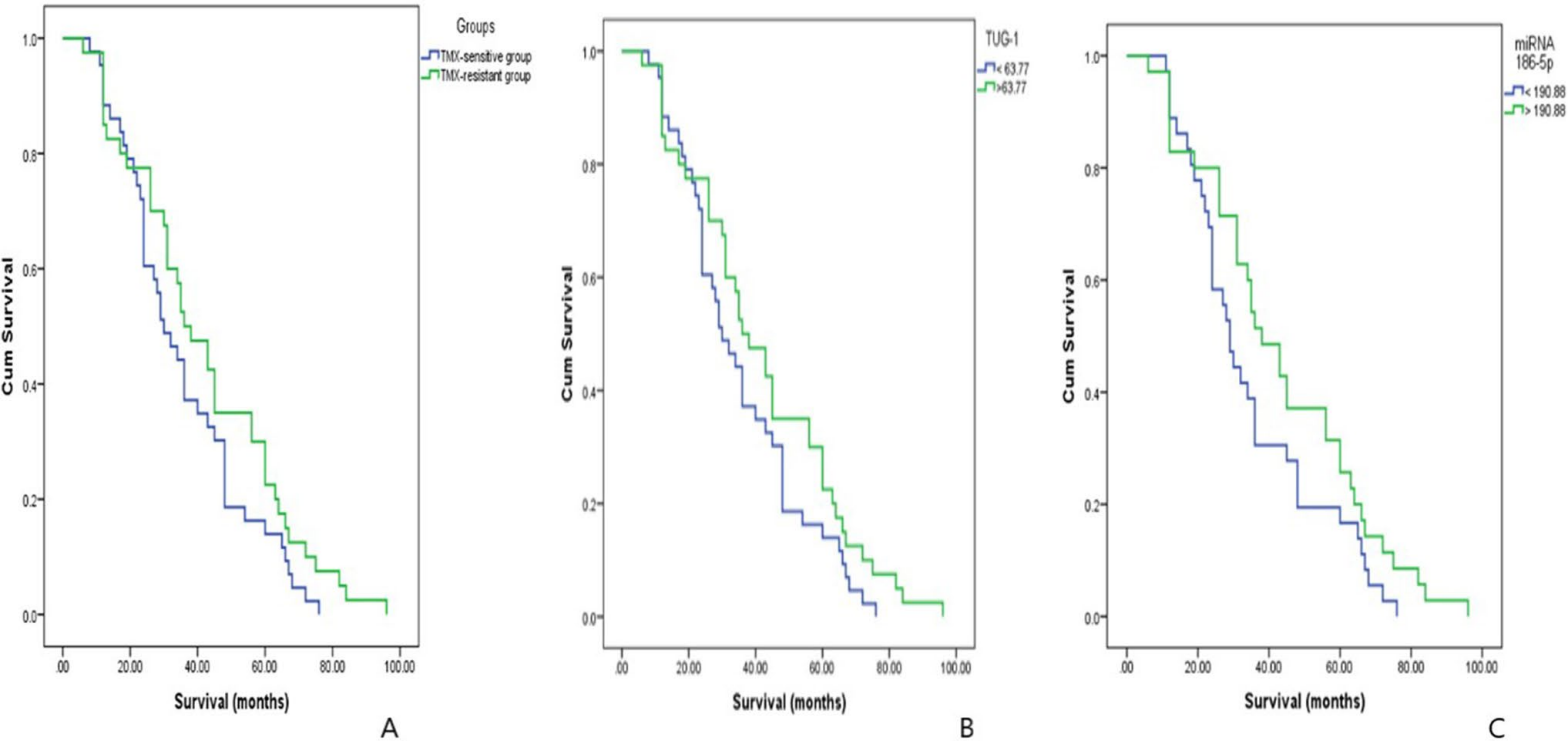
Kaplan–Meier analysis. In this figure, Kaplan–Meier analysis was used to assess the relation between different parameters and survival (months) and also to compare between resistant and sensitive groups using Log Rank test. (**A**) Comparison between TMX-sensitive and TMX-resistant groups regarding overall survival (months) shows that there was no statistical significant difference found between TMX-sensitive group and TMX-resistant group regarding overall survival with P value = 0.117. (**B**) The relationship between TUG-1 with survival (months) shows that there was no statistical significant difference found in TUG-1 regarding overall survival with P value = 0.117. (**C**) The relationship between miR-186 with survival (months) shows that there was no statistical significant difference found in miR-186 regarding overall survival with P value = 0.114.

Using Kaplan–Meier analysis, Fig. 3A shows clearly that there was no statistically significant difference found between TMX-sensitive group and TMX-resistant group regarding overall survival with P value = 0.117. Figure 3B shows that there was no statistically significant difference found in TUG-1 regarding overall survival with P value = 0.117 and Fig. 3C shows that there was no statistically significant difference found in miR-186 regarding overall survival with P value = 0.114.

### Correlations between TUG-1, miRNA 186-5, SIRT3, PPAR-1α and HIF-1α

**Figure 4:**
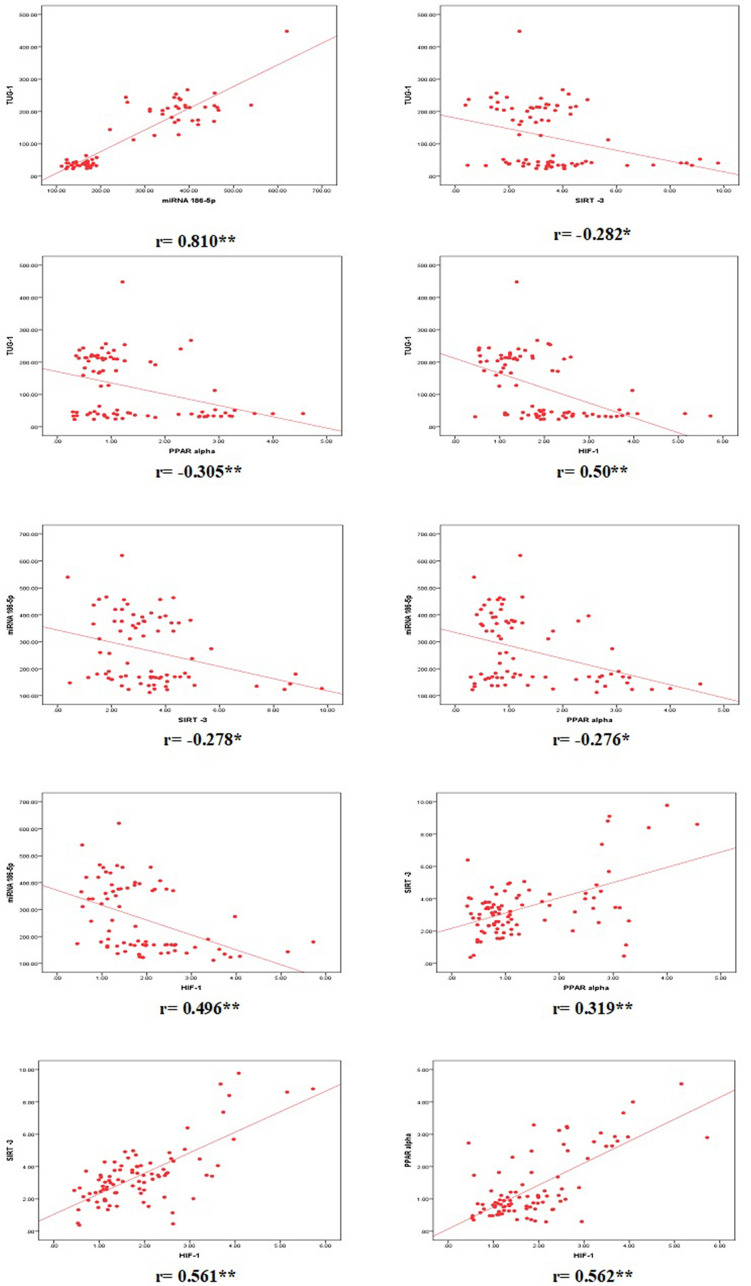
Correlations between TUG-1, miRNA 186-5, SIRT3, PPAR-1α and HIF-1α. In this figure, Spearman correlation coefficients were used to assess the correlation between two quantitative parameters in the same group. Regarding TUG-1, it showed significant positive correlation with miR-186 and significant negative correlation with SIRT-3, PPAR-1 α and HIF-1α. On the other hand, miR-186 showed significant negative correlation with SIRT-3, PPAR-1 α and HIF-1α. Moreover, SIRT-3 showed significant positive correlation with PPAR-1 α and HIF-1α. Last but not least, PPAR-1 α showed significant positive correlation with HIF-1α. R values (correlation coefficients) are shown below each correlation.

Regarding TUG-1, it showed significant positive correlation with miR-186 and significant negative correlation with SIRT3, PPAR-1 α and HIF-1α. On the other hand, miR-186 showed significant negative correlation with SIRT3, PPAR-1 α and HIF-1α. Moreover, SIRT3 showed significant positive correlation with PPAR-1 α and HIF-1α. Last but not least, PPAR-1 α showed significant positive correlation with HIF-1α. Table 4 and Fig. 4 show clearly all possible correlations between the different studied parameters;

**Table 4:** Correlations between the different studied parameters.

	TUG-1		miR-186		SIRT3		PPAR alpha		HIF-1	
	r	P value	r	P value	r	P value	r	P value	r	P value
TUG-1	–	–	0.810	0.000**	− 0.282	0.011*	− 0.305	0.006**	0.50	0.000**
miR-186	0.810	0.000**	–	–	− 0.278	0.014*	− 0.276	0.015*	0.496	0.000**
SIRT3	− 0.282	0.011*	− 0.278	0.014*	–	–	0.319	0.002**	0.561	0.000**
PPAR alpha	− 0.305	0.006**	− 0.276	0.015*	0.319	0.002**	–	–	0.562	0.000**

P value > 0.05: non significant; P value < 0.05: significant*; P value < 0.01: highly significant**.

### Univariate and multivariate logistic regression with TMX resistance

Multivariate analysis in Table 5 shows clearly that only **TUG-1** and **HIF-1α**, revealed statistically significant results at OR of 132.827 and 95% CI of (13.722–1285.766) for TUG-1 and at OR of 28.643 and 95% CI of (2.559–320.580) for HIF-1α, respectively.

**Table 5:** Univariate and multivariate logistic regression analysis.

	Univariate analysis					Multivariate analysis				
	B	S.E	Wald	P value	OR (95% CI)	B	S.E	Wald	P value	OR (95% CI)
TUG-1 > 63.77	4.787	0.798	36.032	0.000**	120.00(25.136–572.889)	**4.889**	**1.158**	**17.818**	0.000**	**132.827 (13.722– 1285.766)**
miR-186 > 243.39	4.225	0.710	35.418	0.000**	68.40 (17.010–275.046)	–	–	–	**ns**	**–**
SIRT3 ≤ 3.39	1.393	0.455	9.390	0.002**	4.028 (1.652–9.819)	–	–	–	**ns**	**–**
PPAR alpha ≤ 1.25	1.959	0.559	12.292	0.000**	7.092 (2.372–21.200)	–	–	–	**ns**	**–**
HIF-1 ≤ 1.46	2.620	0.523	25.074	**0.000**	13.739 (4.926–38.313)	**3.355**	**1.232**	**7.412**	0.006**	**28.643 (2.559–320.580)**

*ns* non-significant.

Significant values are in [bold].

### Receiver operating characteristic curve for the best cut off point between TMX-sensitive and TMX-resistant groups regarding TUG-1 and HIF-1α

ROC curve for the best cut off point between sensitive and resistant groups regarding different parameters can be seen clearly in Table 6. This was done for multivariate significance in order to establish an experimental model for our study.

**Table 6:** ROC table for the best cut off point between sensitive and resistant groups regarding TUG-1 and HIF-1α.

Variables	Cut off point	AUC	Sensitivity	Specificity	+ PV	− PV
TUG-1	** > 63.77**	0.995	97.5	97.7	97.5	97.7
HIF-1	** ≤ 1.46**	0.815	73.81	82.98	79.5	78.0
Combined	–	0.946	88.37	91.67	90.5	89.8

**Figure 5:**
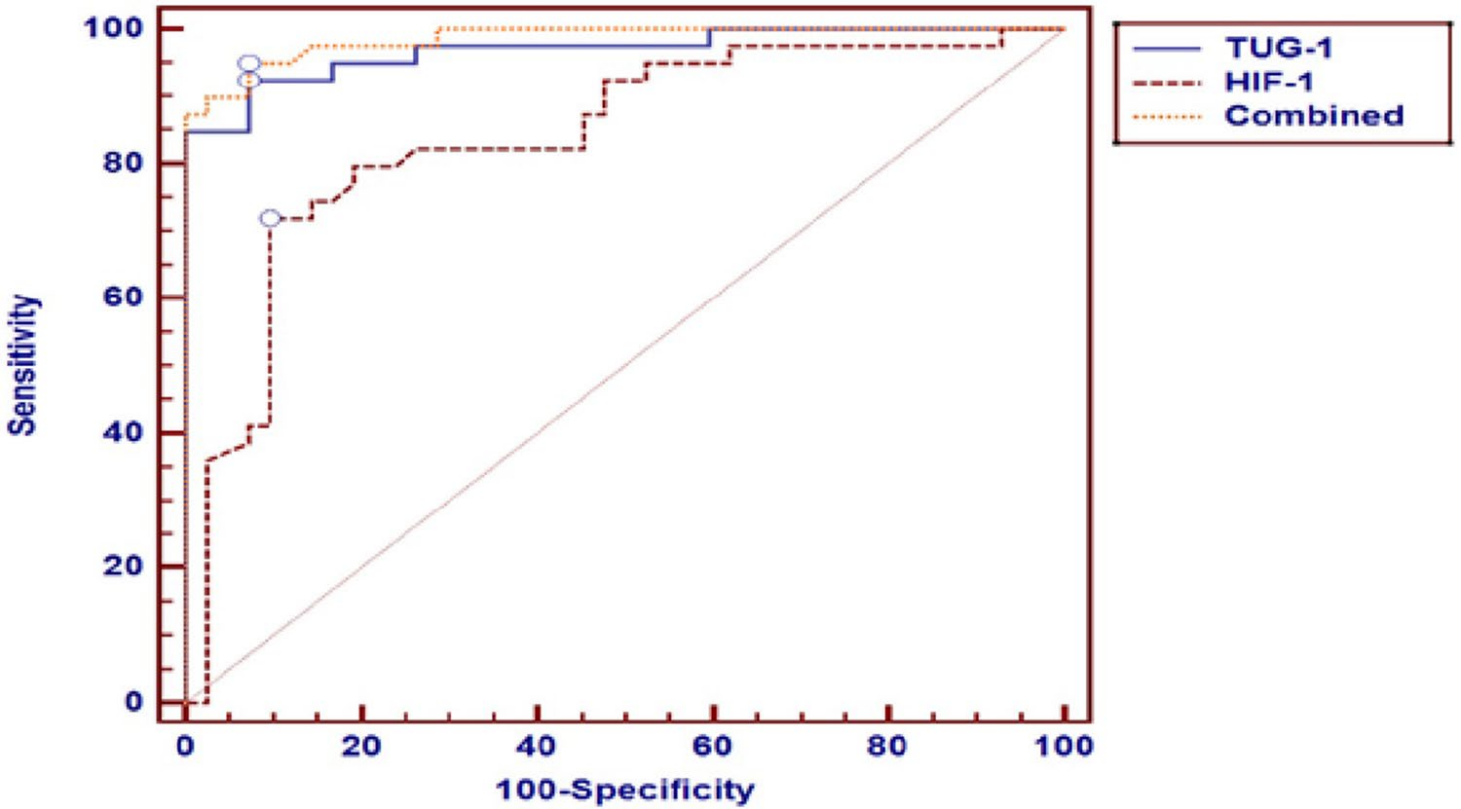
ROC for the best cut off point between TMX-sensitive and TMX-resistant groups regarding TUG-1 and HIF-1α. ROC curve was used to assess the best cut off point for predictors of mortality with its sensitivity, specificity, positive predictive value, negative predictive value and area under curve. Logistic regression analysis (univariate and multivariate) was used to assess the association between symptoms and different parameters with its odds ratio and 95% CI. This figure was constructed for multivariate significance in order to establish an experimental model for our study (with 88.3% sensitivity and 91.6% specificity), where BC patients with TUG-1 value > 63.77 and HIF-1α value < 1.46 can be considered resistant to TMX treatment.

In other words, cut-off values for TUG-1 and HIF-1α can be used in the ROC model for determining TMX resistance BC patients. BC patients have a **TUG-1 cut off value of 63.77**. Furthermore, they have a **HIF-1α cut off value of 1.46**. This is shown in Fig. 5.

## Discussion

Exploring the mechanism of TMX resistance in BC patients is a must as it is still far from clear. Besides, there is an unmet need to find a reliable biomarker for TMX resistance in BC patients. Knowing that metabolic dysregulations and hypoxic involvements are strongly introduced as one of the main factors of TMX resistance^11^, this study aimed at exploring the possible role of the metabolic/hypoxial axis TUG-1/miR-186/SIRT3, PPAR-1α, HIF-1α in TMX sensitive and resistant BC patients as it might be a crucial link in understanding metabolic rewiring on TMX resistance and might be used as a future biomarker.

TMX acts as an ER antagonist for the breast, and its selective actions on tissues turned it to be the most significant treatment for ER-positive BC. There have been several studies revealing efficacy for the treatment and prevention of ER-positive BC. TMX is mainly cytostatic and acts by slowing down the proliferation of BC cells by hindering their progression from the G1 phase of the cell cycle^34^.

In the biology of rapidly growing tumors, metabolic demand is often upregulated, this necessitates a more-efficient energy source for continuous growth. Although aerobic glycolysis has been surmised as a crucial characteristic of cancer cells for years, cancer studies provide evidence that mitochondrial respiration has critical roles in tumorigenesis^35^. Several groups have circulated that TMX is able to directly alter mitochondrial function. It preferentially accumulates in cellular membranes and its accumulation within mitochondria impacts crucial processes such as respiration, fatty acid oxidation, mitochondrial DNA (mtDNA) synthesis and replication, and expression of mitochondrially encoded subunits of electron transport chain^36,37^.

Metabolic pathways or processes connected with mitochondria such as glucose metabolism, lipogenesis, amino-acid metabolism, and nucleotide biosynthesis are found to intersect with tumor progression^38^. Mitochondria generate oncogenic metabolites, which can alter epigenetic states of cancer cells. Moreover, mitochondria also generate reactive oxygen species (ROS), which facilitate deoxyribonucleic acid (DNA) mutations and tumor progression^39^.

When comparing TUG-1 levels in TMX-sensitive and TMX-resistant groups, it was found that plasma levels of TUG-1 were significantly elevated in TMX resistant patients compared to age-matched TMX sensitive patients. TUG-1 has been demonstrated to be enhanced in BC^40^*.* Furthermore, it was abnormally regulated in tumorigenesis, either as a potential tumor suppressor or as an oncogene^41^.

Bioinformatics studies found that TUG-1 is involved in gene regulation through several mechanisms; primarily by operating as a miRNA sponge^42,43^. To our knowledge, our study is the first to determine the expression level of miR-186 in TMX sensitive and resistant BC patients. Our results revealed that plasma levels of miR-186 were significantly elevated in TMX resistant patients compared to age-matched TMX sensitive patients. In the same line previous reports showed that miR-186 was upregulated in several cancers, which both enhanced cell proliferation and migration, and inhibited apoptosis by repressing several targets^44^. Our findings proved at least in part an association between respiratory symptoms and miR-186 levels where it showed that respiratory symptoms rate increase as miR-186 decreases in TMX resistant patients. This can be assured by the downregulation of miR-186 expression in non-small cell lung cancer (NSCLC), which is the most common type of respiratory cancers. This forecasts poor patient survival^45–48^.

Similarly, Bentzen et al. identified TMX as a risk factor for radiotherapy-related lung fibrosis^49^. Moreover, a case of TMX-induced lung injury was reported in Japan in a patient who had received radiation therapy^50^. Added to that, miR-186 has been recently reported to control inflammatory fibroblasts via regulating HIF-1α in chronic obstructive pulmonary disease^51^.

miRNAs are mainly post-transcriptional regulators that affect mRNA stability and protein levels which can also modulate SIRT3 activity. Our results revealed that serum levels of SIRT3 were significantly deficient in TMX resistant patients compared to age-matched TMX sensitive patients. Similar associations were found in some studies which establish a tumor suppressor role for SIRT3^15^. In agreement with this, others found that, in BC patients, SIRT3 levels were lower or undetectable in most of the samples than in normal individuals, and specifically breast and ovarian cancers were frequently associated with focal deletion of the SIRT3 gene^52^.

Several studies showed that SIRT3 was a direct, positively regulated target of PPAR-1 α^53^. A significant elevation of PPAR-1 α mRNA expression is seen in mammary gland carcinomas, raising the possibility that PPAR-1 α has a role in mammary gland carcinogenesis^54^. Our results manifested that serum levels of this protein were significantly deficient in TMX resistant patients compared to age-matched TMX sensitive patients. Given the role of PPAR-1 α in regulating gene expression, including those involved in lipid homeostasis^55^, and given the relationship between PPAR-1 α and activation of the prolactin gene^56^, it is likely that PPAR-1 α has a role in both the normal and the tumorigenic mammary gland.

One of the most defining features of solid tumors is hypoxia caused by an inadequate supply of oxygen. Low oxygen levels incite changes in cancer cells and in other components of the tumor microenvironment. This can be attributed to HIFs, helping in maximizing the metastatic potential of cancer cells^57^. Various studies displayed a correlation between hypoxia and carcinogenesis, metastasis, treatment failure, and patient mortality^58,59^.

It was previously shown that ERα-positive BC cells grown under hypoxic conditions were resistant to antiestrogens as TMX, while they were sensitive to treatment at normoxia^60^. Our results highlighted the enhancement in metastases rate as HIF-1 increases in TMX resistant candidates. Relevant to this, the activation of the hypoxia pathway in BC has been shown to play a crucial role by aiding in processes like the formation of new blood vessels (angiogenesis), remodeling of the extracellular matrix, and establishment of pre-metastatic pool, invasion and extravasation at metastatic site. Previous studies have reported changes in alternative splicing due to hypoxia in BC cells^57^.

Plasma miR-186 showed significant direct correlation with plasma TUG-1 levels. This agrees with previous studies which showed that TUG-1 mediates chemoresistance in colorectal cells by sponging miR-186 and easing the oppression of miR-186 on oncogenic proteins^61^. A recent study revealed that TUG-1 acts as a ceRNA that prevents downregulation of SIRT3^40^. TUG-1 also has been found to be up-regulated in oxygen-deprived myocardial cells^62^. This dovetails with our results which showed clear inverse correlations between TUG-1 and SIRT3 and between TUG-1 and HIF-1 α, respectively. Of concern, miRNAs are mainly post-transcriptional regulators that affect mRNA stability and protein levels which can also regulate SIRT3 activity^63^.

Furthermore, Previous studies in cancer reported that HIF-1 α could act as the target for miR-186^64^*.* This crosslinks with the significant inverse correlations found between miR-186 and SIRT3 and between miR-186 and HIF-1 α, respectively. Studies showed that SIRT3 was a direct, positively regulated target of PPAR-1 α and a downstream target of PPAR-1 α^53^*.* From these correlations, we can conclude that all parameters are targets for each other, consequently, we constructed backward regression analysis to find the highest target values. Moreover, strong correlation between HIF-1 and other markers assured the potential association between hypoxia and metabolism. Thus, those markers can be used as reliable targets in the future.

SIRT3 stimulates greater HIF-1α protein stabilization in hypoxic conditions claiming that SIRT3 regulates HIF through ROS-mediated alteration of proline hydroxylation enzyme function, thereby altering hydroxylation and upcoming proteosomal degradation of HIF-1alpha^65^. This correlates with the significant associations between SIRT3 and PPAR-1 α and between SIRT3 and HIF-1 α, respectively. A significant direct correlation was seen between serum HIF-1 levels and serum PPAR-1 α levels. This indicates that HIF-1-dependant down-regulation of PPAR-1 α may furnish an adaptive response to proinflammatory stimuli during cellular hypoxia. These studies provide unique insight into the regulation of PPAR-1 α expression and, importantly, provide an example of a down-regulatory pathway mediated by HIF-1^66^.

Summing up, targeting the HIF pathway might provide an attractive strategy to treat hypoxic tumors. For instance, HIF-1 inhibitors, such as digoxin and acriflavine, showed convincing potential therapeutic effects by diminishing primary tumor growth, vascularization, invasion- and metastasis in BC animal models^67^. As for ER-positive BC patients, hypoxia has been shown to down-regulate ERα in several BC cell lines and to influence the responsiveness to TMX^68^. Relevant to this, our results showed that low HIF-1 α levels cause ER up-regulation and thus TMX resistance.

Simultaneously, SIRT3 could sustain ROS homeostasis through the regulation of a complex of mitochondrial enzymes such as superoxide dismutase 2, which may change harmful superoxide radicals into nontoxic oxygen or hydrogen peroxide^69^. Our results conveyed that SIRT3 levels are significantly lower in TMX resistant patients and this enhances ROS and oxidative stress. This causes tumor cells growth.

PPAR-1α is a transcriptional regulator of genes involved in peroxisomal and mitochondrial beta-oxidation^70^. These findings dovetail with our results, which proposed that TMX resistant patients have lower PPAR-1α levels. This diminishes beta-oxidation and enhances lipogenesis and tumor growth. Moreover, PPAR-1α is responsible for fat mobilization during fasting and activates mitochondrial and peroxisomal fatty acid oxidation and ketogenesis, resulting in inhibition of glycolysis and fatty acid synthesis^71^. Glycolysis is enhanced in activated lung fibroblasts and upregulated in macrophages from fibrotic lung tissues^72^. This crosslink with our results, which highlighted low PPAR-1α levels and miR-186 levels as glycolysis stimulants, which in turn result in worser respiratory outcomes in TMX-resistant group.

Our study showed at least in a part that TUG-1 and HIF-1 α were significantly associated with TMX resistance in multi-variate logistic regression analysis with 88.3% sensitivity and 91.6% specificity. In other words, cut-off values for TUG-1 and HIF-1α can be used in the ROC model for determining TMX resistance BC patients. BC patients have a **TUG-1 cut off value of 63.77**. Furthermore, they have a **HIF-1α cut off value of 1.46.**

**Figure 6:**
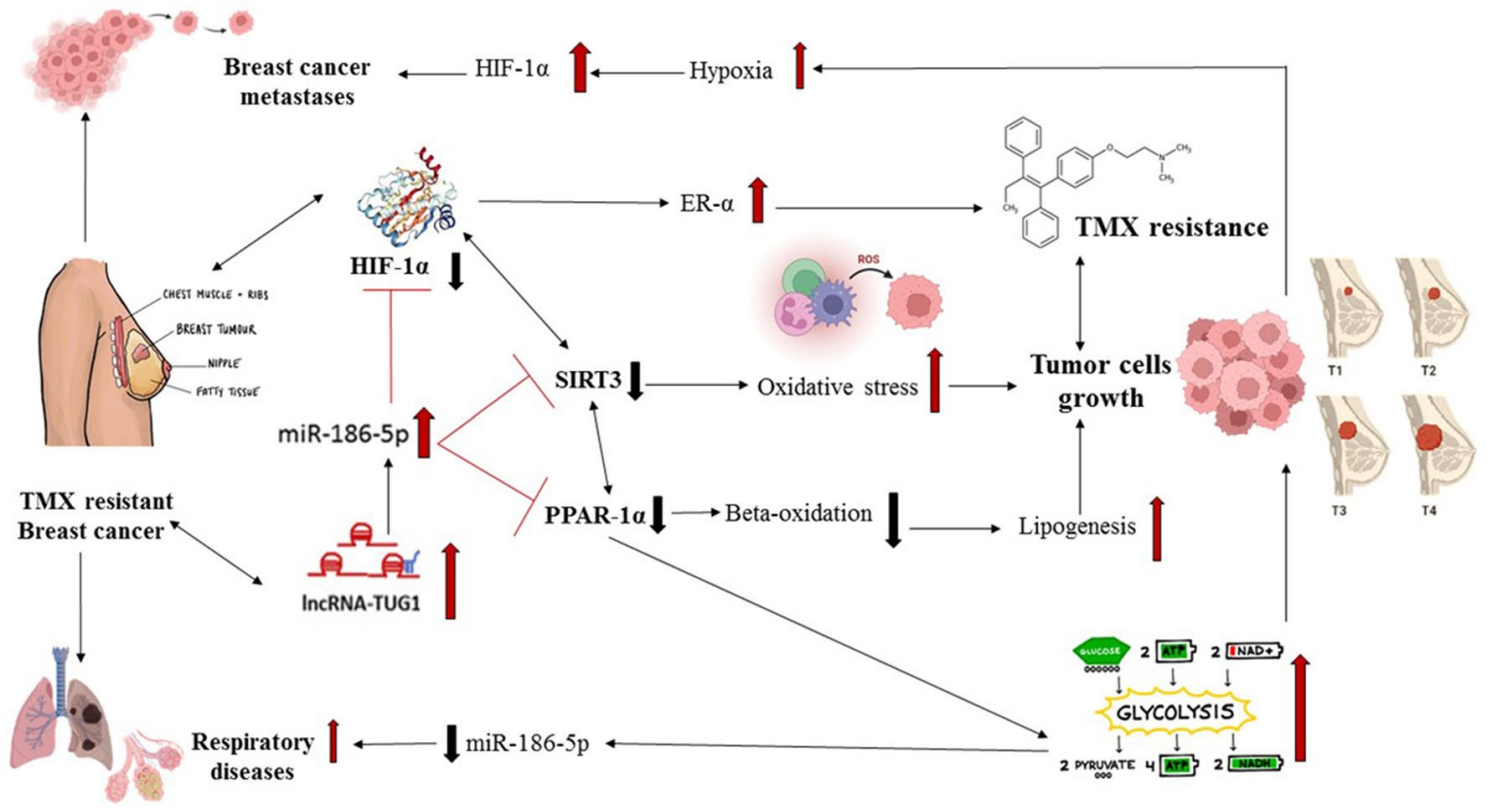
Low HIF-1α levels increased ER expression and in turn TMX resistance^68^. The decrease in SIRT3 have resulted in higher levels of oxidative stress and thus enhanced tumor cells growth^69^. Moreover, diminished levels of PPAR-1 α have led to lower levels of beta oxidation which ended up in lipogenesis (tumor cells growth) and in higher glycolysis levels and consequently lower miR-186 levels and enhancement in respiratory disease^70–72^. Summing up, the aftermath of tumor cells growth settled high levels of HIF-1α (hypoxia) and BC metastases.

This model highlights the importance of metabolic-hypoxial axis to avoid TMX resistance in BC patients and increase the success rates (Fig. 6). It is pivotal to explore the decisive influence of TMX resistance on the course of BC in order to gain a deeper understanding of individual disease trajectories and to better forecast them.

## Supplementary Information

Supplementary Information.

## Data Availability

Authors can confirm that all relevant data are included in the article and/or its Supplementary Information files.

## Abbreviations

AUC: Area under curve
BC: Breast cancer
BMI: Body mass index
ceRNA: Competing endogenous RNA
CT: Threshold cycle
DM: Diabetes mellitus
DNA: Deoxyribonucleic acid
ELISA: Enzyme linked immunosorbent assay
EMT: Epithelial‑mesenchymal transition
ER: Estrogen
ETC: Electron transport chain
GAPDH: Glyceraldehyde-3-phosphate dehydrogenase
HIF-1 α: Hypoxia inducible factor-1
IBM SPSS: Statistical Package for Social Science
IQR: Inter-quartile range
LncRNAs: Long-noncoding RNAs
miR-186: MiRNA 186-5p
MiRNAs: MicroRNAs
Ml: Milliliters
MtDNA: Mitochondrial DNA
NPV: Negative predictive value
NSCLC: Non-small cell lung cancer
OR: Odd ratio
CI: Confidence interval
PPAR-1 α: Peroxisome proliferator activator receptor alpha
r.p.m: Round per minute
RBCs: Red blood cells
ROC: Receiver operating characteristic curve
ROS: Reactive oxygen species
RT-PCR: Real-time polymerase chain reaction
SIRT3: Sirtuin-3
TMX: Tamoxifen
TUG-1: Taurine upregulated-1

## References

[1] Hultsch S (2018). Association of tamoxifen resistance and lipid reprogramming in breast cancer. BMC Cancer.

[2] Jiang N (2021). Progress in understanding the role of lncRNA in programmed cell death. Cell Death Discov..

[3] Lopez-Pajares V (2016). Long non-coding RNA regulation of gene expression during differentiation. Pflugers Arch..

[4] Guo X, Hua Y (2017). CCAT1: An oncogenic long noncoding RNA in human cancers. J. Cancer Res. Clin. Oncol..

[5] Zhou H, Sun L, Wan F (2019). Molecular mechanisms of TUG1 in the proliferation, apoptosis, migration and invasion of cancer cells. Oncol. Lett..

[6] Al-Asmari AK (2016). Taurine ameliorates 5-flourouracil-induced intestinal mucositis, hepatorenal and reproductive organ damage in Wistar rats: A biochemical and histological study. Hum. Exp. Toxicol..

[7] Guo C (2020). Pathophysiological functions of the lncRNA TUG1. Curr. Pharm. Des..

[8] Che X, Qian Y, Li D (2018). Suppression of disheveled-axin domain containing 1 (DIXDC1) by microRNA-186 inhibits the proliferation and invasion of retinoblastoma cells. J. Mol. Neurosci..

[9] Sun WJ, Zhang YN, Xue P (2018). miR-186 inhibits proliferation, migration, and epithelial-mesenchymal transition in breast cancer cells by targeting Twist1. J. Cell. Biochem..

[10] Mulrane L (2013). miRNA dysregulation in breast cancer. Cancer Res..

[11] Tomková V (2019). Mitochondrial fragmentation, elevated mitochondrial superoxide and respiratory supercomplexes disassembly is connected with the tamoxifen-resistant phenotype of breast cancer cells. Free Radic. Biol. Med..

[12] Desouki MM (2014). Decreased mitochondrial SIRT3 expression is a potential molecular biomarker associated with poor outcome in breast cancer. Hum. Pathol..

[13] Haigis MC (2012). SIRT3 is a mitochondrial tumor suppressor: A scientific tale that connects aberrant cellular ROS, the Warburg effect, and carcinogenesis. Cancer Res..

[14] Kong X (2010). Sirtuin 3, a new target of PGC-1alpha, plays an important role in the suppression of ROS and mitochondrial biogenesis. PLoS One.

[15] Finley LW (2011). SIRT3 opposes reprogramming of cancer cell metabolism through HIF1α destabilization. Cancer Cell.

[16] Harris AL (2002). Hypoxia—a key regulatory factor in tumour growth. Nat. Rev. Cancer.

[17] Patterson B, Guthrie C (1987). An essential yeast snRNA with a U5-like domain is required for splicing in vivo. Cell.

[18] Boom R (1990). Rapid and simple method for purification of nucleic acids. J. Clin. Microbiol..

[19] Saiki RK (1985). Enzymatic amplification of beta-globin genomic sequences and restriction site analysis for diagnosis of sickle cell anemia. Science.

[20] Mullis KB, Faloona FA (1987). Specific synthesis of DNA in vitro via a polymerase-catalyzed chain reaction. Methods Enzymol..

[21] Kwok S, Higuchi R (1989). Avoiding false positives with PCR. Nature.

[22] Benedict C (2012). Acute sleep deprivation has no lasting effects on the human antibody titer response following a novel influenza A H1N1 virus vaccination. BMC Immunol..

[23] Livak KJ, Schmittgen TD (2001). Analysis of relative gene expression data using real-time quantitative PCR and the 2(-Delta Delta C(T)) method. Methods.

[24] Lim LP (2003). The microRNAs of *Caenorhabditis elegans*. Genes Dev..

[25] Bartel DP (2004). MicroRNAs: Genomics, biogenesis, mechanism, and function. Cell.

[26] Kim J (2004). Identification of many microRNAs that copurify with polyribosomes in mammalian neurons. Proc. Natl. Acad. Sci. USA.

[27] Cooper HM, Spelbrink JN (2008). The human SIRT3 protein deacetylase is exclusively mitochondrial. Biochem. J..

[28] Schlicker C (2008). Substrates and regulation mechanisms for the human mitochondrial Sirtuins Sirt3 and Sirt5. J. Mol. Biol..

[29] Cronet P (2001). Structure of the PPARalpha and -gamma ligand binding domain in complex with AZ 242; ligand selectivity and agonist activation in the PPAR family. Structure (Lond., Engl.: 1993).

[30] Xu HE (2002). Structural basis for antagonist-mediated recruitment of nuclear co-repressors by PPARalpha. Nature.

[31] Denizot J (2012). Tu1908 HIF-1a regulates CEACAM6 expression in response to Crohn's disease-associated *E. coli* infection in a DNA methylation dependent manner. Gastroenterology.

[32] Arslan F (2021). Evaluation of potential tumor markers that may predict neoadjuvant treatment efficiency in rectal cancer. Turk. J. Biochem..

[33] Lin PC (2016). Long noncoding RNA TUG1 is downregulated in non-small cell lung cancer and can regulate CELF1 on binding to PRC2. BMC Cancer.

[34] Riggs B, Hartmann L (2003). Selective estrogen-receptor modulators—mechanisms of action and application to clinical practice. N. Engl. J. Med..

[35] Israelsen WJ, Vander Heiden MG (2015). Pyruvate kinase: Function, regulation and role in cancer. Semin. Cell Dev. Biol..

[36] Voss G (2018). Progress and challenges in TB vaccine development. F100Res.

[37] Fiorillo M (2015). Mitochondrial “power” drives tamoxifen resistance: NQO1 and GCLC are new therapeutic targets in breast cancer. Oncotarget.

[38] Ahn CS, Metallo CMJC (2015). Mitochondria as biosynthetic factories for cancer proliferation. Cancer Metab..

[39] Sullivan LB, Gui DY, Vander Heiden MGJNRC (2016). Altered metabolite levels in cancer: Implications for tumour biology and cancer therapy. Nat. Rev. Cancer.

[40] Zeng B (2017). LncRNA TUG1 sponges miR-145 to promote cancer progression and regulate glutamine metabolism via Sirt3/GDH axis. Oncotarget.

[41] Li J (2016). LncRNA TUG1 acts as a tumor suppressor in human glioma by promoting cell apoptosis. Exp. Biol. Med. (Maywood).

[42] Lin P-C (2016). Long noncoding RNA TUG1 is downregulated in non-small cell lung cancer and can regulate CELF1 on binding to PRC2. BMC Cancer.

[43] Khalil AM (2009). Many human large intergenic noncoding RNAs associate with chromatin-modifying complexes and affect gene expression. Proc. Natl. Acad. Sci..

[44] Xiang Y (2020). The dual role of miR-186 in cancers: Oncomir battling with tumor suppressor miRNA. Front. Oncol..

[45] Huang T (2016). MicroRNA-186 suppresses cell proliferation and metastasis through targeting MAP3K2 in non-small cell lung cancer. Int. J. Oncol..

[46] Dong Y (2017). MiR-186 inhibited migration of NSCLC via targeting cdc42 and effecting EMT process. Mol. Cells.

[47] Huang T (2017). MiR-186 inhibits proliferation, migration, and invasion of non-small cell lung cancer cells by downregulating Yin Yang 1. Cancer Biomark..

[48] Ruan L (2018). MicroRNA-186 suppresses lung cancer progression by targeting SIRT6. Cancer Biomark..

[49] Bentzen SM (1996). Radiotherapy-related lung fibrosis enhanced by tamoxifen. J. Natl. Cancer Inst..

[50] Shiiki SJNRGGZ (2003). A case of drug-induced pneumonia due to tamoxifen. Nihon Rinsho Geka Gakkai Zasshi (J. Jpn. Surg. Assoc.).

[51] Lin L (2019). MicroRNA-186 is associated with hypoxia-inducible factor-1α expression in chronic obstructive pulmonary disease. Mol. Genet. Genom. Med..

[52] Bell EL (2011). SirT3 suppresses hypoxia inducible factor 1α and tumor growth by inhibiting mitochondrial ROS production. Oncogene.

[53] Zong X (2020). SIRT3 is a downstream target of PPAR-α implicated in high glucose-induced cardiomyocyte injury in AC16 cells. Exp. Ther. Med..

[54] Roberts-Thomson S, Snyderwine E (2001). Characterization of peroxisome proliferator-activated receptor alpha in normal rat mammary gland and 2-amino-1-methyl-6-phenylimidazo[4,5-b]pyridine-induced mammary gland tumors from rats fed high and low fat diets. Toxicol. Lett..

[55] Schoonjans K, Staels B, Auwerx J (1996). The peroxisome proliferator activated receptors (PPARs) and their effects on lipid metabolism and adipocyte differentiation. Biochim. Biophys. Acta Lipids Lipid Metab..

[56] Tolón RM, Castillo AI, Aranda A (1998). Activation of the prolactin gene by peroxisome proliferator-activated receptor-alpha appears to be DNA binding-independent. J. Biol. Chem..

[57] Pant D (2020). Hypoxia-induced changes in intragenic DNA methylation correlate with alternative splicing in breast cancer. J. Biosci..

[58] Rankin EB, Nam JM, Giaccia AJ (2016). Hypoxia: Signaling the metastatic cascade. Trends Cancer.

[59] Semenza GL (2016). (2016) The hypoxic tumor microenvironment: A driving force for breast cancer progression. Biochim. Biophys. Acta Mol. Cell Res..

[60] Alam MW (2016). HIF2α contributes to antiestrogen resistance via positive bilateral crosstalk with EGFR in breast cancer cells. Oncotarget.

[61] Li C (2017). TUG1 mediates methotrexate resistance in colorectal cancer via miR-186/CPEB2 axis. Biochem. Biophys. Res. Commun..

[62] Wu Z (2018). LncRNA TUG1 serves an important role in hypoxia-induced myocardial cell injury by regulating the miR-145-5p-Binp3 axis. Mol. Med. Rep..

[63] Zhang J (2020). Mitochondrial Sirtuin 3: New emerging biological function and therapeutic target. Theranostics.

[64] Liu L (2016). MiR-186 inhibited aerobic glycolysis in gastric cancer via HIF-1α regulation. Oncogenesis.

[65] Sundaresan NR (2009). Sirt3 blocks the cardiac hypertrophic response by augmenting Foxo3a-dependent antioxidant defense mechanisms in mice. J. Clin. Investig..

[66] Narravula S, Colgan SP (2001). Hypoxia-inducible factor 1-mediated inhibition of peroxisome proliferator-activated receptor alpha expression during hypoxia. J. Immunol..

[67] Wong CC (2011). Hypoxia-inducible factor 1 is a master regulator of breast cancer metastatic niche formation. Proc. Natl. Acad. Sci. USA.

[68] Kronblad A (2005). ERK1/2 inhibition increases antiestrogen treatment efficacy by interfering with hypoxia-induced downregulation of ER??: A combination therapy potentially targeting hypoxic and dormant tumor cells. Oncogene.

[69] Bause AS, Haigis MC (2013). SIRT3 regulation of mitochondrial oxidative stress. Exp. Gerontol..

[70] Xu J (2002). Peroxisome proliferator-activated receptor alpha (PPARalpha) influences substrate utilization for hepatic glucose production. J. Biol. Chem..

[71] Frederiksen KS (2004). Prediction of PPAR-alpha ligand-mediated physiological changes using gene expression profiles. J. Lipid Res..

[72] Xie N (2017). Metabolic characterization and RNA profiling reveal glycolytic dependence of profibrotic phenotype of alveolar macrophages in lung fibrosis. Am. J. Physiol. Lung Cell Mol. Physiol..

